# Clinical diagnosis of cardiac involvement in HIV infection

**Published:** 2012-09-25

**Authors:** L Moldovan, O Branzan, O Nechita, C Ardeleanu, M Teodorescu, A Geamai

**Affiliations:** * Cardiology Department, "Th. Burghele" Clinical Hospital, Bucharest, Romania; ** Ministry of Health, Romania; *** "V. Babes" Morpho-Pathology Institute, Bucharest; **** "V. Babes" Clinical Hospital of Infectious and Tropical Disease, Bucharest

**Keywords:** HIV, immunological diagnosis, clinical diagnosis, sinus tachycardia

## Abstract

HIV infection is continuously raising, and different treatments did not manage to extend the patient's life. Clinical and morphopathological features of respiratory, gastrointestinal, hematological and nervous system are well characterized in HIV infection, but cardiac involvement is not so well known.

Cardiac involvement is extremely rare in HIV disease, but demonstrated by echocardiography and anatomo-pathologic methods, it is more frequently met than the clinical features are supposed to be, and it can be demonstrated by positive serologic tests.

The main reason of this research is the necessity to obtain data from HIV infection concerning heart involvement.

## Methodology

The clinical study was done on 60 patients with HIV infection in different stages of the disease, treated with antiretroviral therapy. In order to have a comparison, 19 patients – witness group, without HIV infection, were included in the study. This study included HIV infected patients, one of them hospitalized (15 patients) or treated and supervised in ambulatory, during a 24 hour hospitalization (45 patients).The study was done by following the immunological and clinical parameters.

**Fig. 1:**
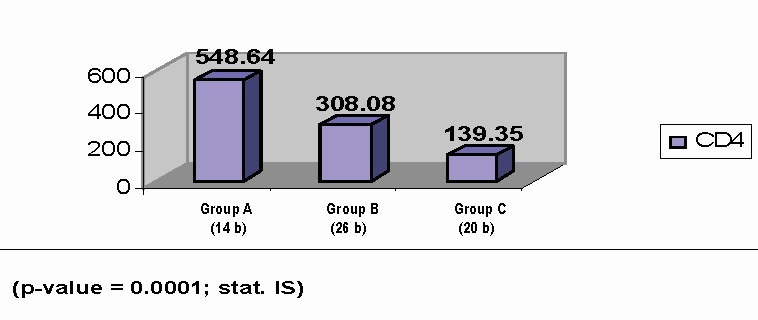
The distribution and comparison of the medium values of CD4 between the groups, in the study lot

Immunological investigations consisted in umoral and cellular immunity research. The normal number of CD4 lymphocytes is between 500 and 1500/mm³. The mean value of CD4 lymphocytes for HIV infected patients is of 307,96 mm³. The mean number of CD4 lymphocytes in the 3 groups was (**Fig. 1**) the following:

A group: 548, 64±208, 72 mm³

B group: 308, 08 ±206, 56 mm³

C group: 139, 35±167, 45 mm³

The differences between the mean value of CD4 lymphocytes in the 3 groups were statistically important (p=0, 0001). Increased levels of CD4 lymphocytes in some of the patients from the C class, with levels over 200/mm³ have the significance of an efficient antiretroviral treatment. So, for the C group, the mean value of CD4 lymphocytes is X=149, 6/mm³. Severe immunodeficiency, with values under 200/mm³ is associated with cardiac complications in these patients. According to the literature, we can appreciate the risk of progression to AIDS, in the next 3 years, depending on the number of CD4 lymphocytes.

**Fig. 2:**
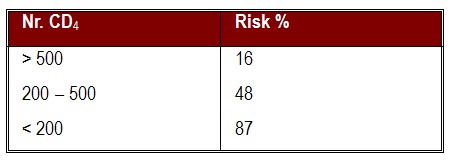
The number of CD4 lymphocytes and the risk of progression to AIDS

CD4 lymphocytes under 100/mm³ are identified as being an important risk factor in cardiac development in HIV infection [**19**].

An African study [**10,11**] shows that the higher the number of CD4 lymphocytes, the lower the progression of the disease, the cardiomyopathy in HIV infection being associated with longer survival rates.

The risk of cardiac complications rises with therapeutic progression [**4**]. HIV infection induces a slow decrease of cholesterol and late rise of triglycerides, with HDL decreasition. These changes are related to the number of CD4 lymphocytes, which reveals the severity of the infection. In a study in 1995, Currie P [**3**], appreciated that it is a thigh connection between dilatative cardiomyopathy and the decrease of CD4 lymphocytes that reach less than 100mm³.

Clinic-immunological features:

Scoping the cases in different evolution stages of HIV infection was done according to the clinical and immunological features of adult classification disease (CDC-1993) in the ''Classification system of HIV infection'' and ''Extended definition of monitoring the AIDS case'' (CDC-1993). Clinic-immunological classification of the 60 patients infected with HIV, from the study, was the following:

- A group -14 patients (23,3%)

- B group - 26 patients (33,3%)

- C group - 20 patients (26%)

**Fig. 3:**
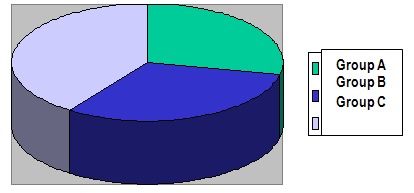
Clinic-immunological classification

The followed clinical parameters were sex, age, weight, blood pressure, cardiac frequency and signs of myocardial, pericardial and cardiac function.

The demographic aspects of patients with HIV infection were:

**a) AGE:** The HIV infected patients’ age was between 17 and 79 years old, the mean age being of 35, 48 years old. The patients' age from the witness group was of 23 and 71 years old, the mean being X=39, 63 years old.

**b) SEX:** The patient's sex from the witness and from the studied group showed a slightly predisposition of females. In HIV infected patients, we studied 23 men and 37 female. Statistically, the differences between the two groups are not important, neither are the differences between sexes. According to the Ministry of Health, HIV infection reveals an equal distribution regarding gender, in Romania.

**Fig. 4:**
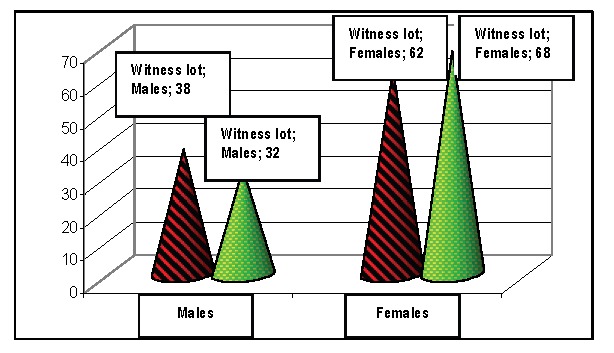
Distribution according to gender

**Fig. 5:**
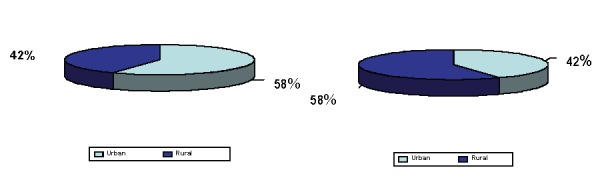
The structure of the lots according to the environment (U/R)

**c) ENVIROMENT:** Big parts of the patients with AIDS come from the urban places (35 patients - 58% versus 25 patients-42% from rural space). The distribution of the patients according to the environment is shown in **Fig. 5**. The difference urban/rural is not statistically important.

It is important to notice that, at the beginning of the report of HIV infection in Romania, in 1985-1989, only one case existed in the rural environment, the rest being registered in the urban places. In the next years, starting with 1990, a higher amount of HIV infected patients was reported also in the rural environment, being as high as the one in the urban place.

**d. HEIGHT:** In the HIV infected group of patients, the height was measured in centimeters, comparative with the studied group. The comparison was done between A, B, C groups of HIV infected patients and the specialty literature. For the specialty literature data from ''F. Rainer'' Anthropological Research Center, Bucharest, were used, according to the mean height in Romania. The 5 figures show some kind of comparison. The height comparison between A, B, C group is not important from the statistical point of view.

**Fig. 6:**
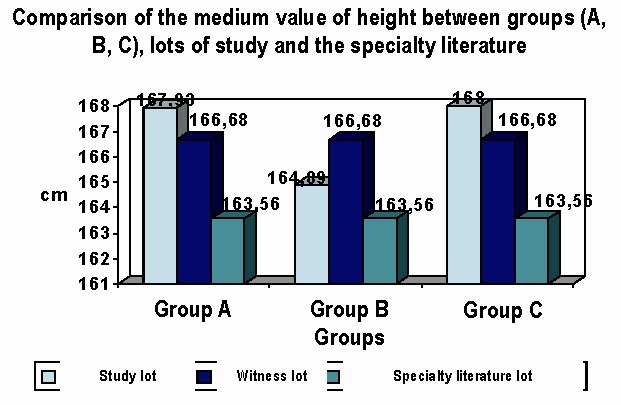
Differences between the groups and lots: insignificant

**e) WEIGHT:** in patients infected with HIV was measured in kilograms; the height was also measured in the witness group. Values in kilograms of the witness and studied group were reported to the normal value of adult weight from Romania.

**Fig. 7:**
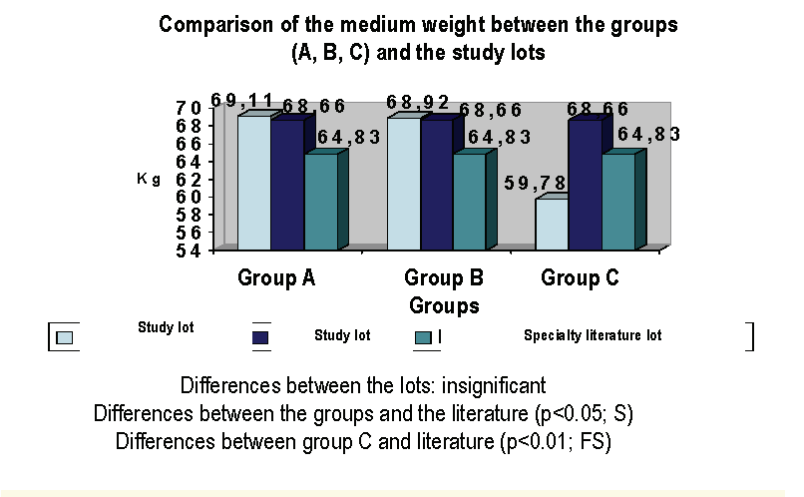
Comparison of the average value of weight in HIV disease groups (A, B, C) between the study group and the witness group, compared with the similar trials on the specialty media

Statistical processing revealed:

• weight compared on HIV groups disease: A, B and C is statistically insignificant.

• weight compared between control group and study group is statistically insignificant

• weight compared between HIV disease groups and dates from specialty literature indicates a difference, statistically significant, with an index of p <0.05. A very significant difference is in HIV infected patients from class C, who have mean values of the weight under those normal values.

Weight deficit is a common clinical manifestation at HIV infected persons, especially at the final of infection [**12**].

Weight deficit is the result of a set of metabolic disturbances in HIV infection, and can be the result of low intake of the basis nutrition, a low absorption of the basis nutrition, a grown energy consumption or losses through diarrhea, vomiting, etc.

**Fig. 8:**
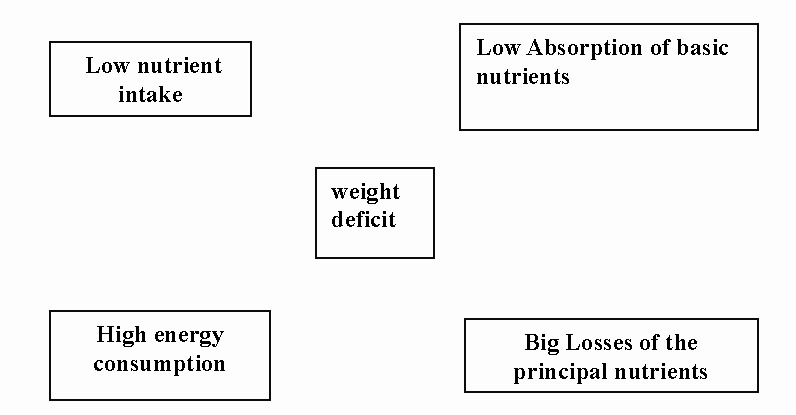
Weight deficit – the result of a set of metabolic disturbances

In a study on HIV infected patients and chronic obstructive pulmonary disease also suffering from malnutrition, authors like Mannix M and E Farber [**5**] appreciate that a link can be made between increased mortality of these patients and their weight decrease, and for these patients, malnutrition physiopathology remains unclear.

We supposed/believed that cardiac cachexia: diet with metabolic factors, cytokine activity level of catecholamine, cortisol, are suspected to be responsible for weight loss in HIV-infected patients.

In **Table 1**, we present the mean values of demographic variables for the HIV infected patients.

**Table 1:** Mean values of the demographic variables in clinical immunological groups of HIV infection

Group variable	A n = 14	B n = 26	C n = 20	F. stat.(Xc2)
Age	33.93±12.74	33.42±12.697	39.25±10.259	Fc = 1.50 p=0.23 (N)
Sex	M=5(35.7%) F=9(64.3%)	M=7(26.9%) F=19(7.31%)	M=11(55%) F=9(45%)	Xc2=3.82 p=0.15 (N)
Medium	U=10(71.4%) R=4(28.6%)	U=10(38.5%) R=16(61.5%)	U=15(75%) R=5(25%)	Xc2=7.5 p=0.02 (S)
CD4	548.64±208.72	308.08±206.56	139.35±167.45	Fc = 18.15 p=10-6 (IS)
height	167.93±7.67	164.89±8.66	168±6.61	Fc = 1.15 p=0.32 (N)
weight	69.11±10.37	68.92±9.43	59.78±6.24	Fc = 7.39 p<0.01 (FS)

Blood pressure

Blood pressure ratings were made by using JNC criteria (Joint National Committee) since 1996.

Elevated blood pressure was observed only in 4 patients (6,66% of cases), aged over 50 years (1 M, 3 F), who are part of Class A - 1 in Class B - 1 case, class C - two cases. All the patients had hypertension before the onset of HIV infection.

Hypotension was observed in 8 patients, mostly young people, the average age being 35 years. (4 M, 4 F). **Table 2**.

From the total 8 hypotensive patients: 4 cases were in class C of HIV infection (AIDS), 2 cases in class A asymptomatic - and 2 cases in class B.

Mean value of the CD4 lymphocytes for all 8 HIV infected patients was of 274,7/mm3.

We believe that the high incidence of hypotension among HIV infected patients, 13,33% of cases, is caused by the severe immunosuppression that is induced by the human immunodeficiency HIV virus, favoring opportunistic infections. 

The etiology of the hypotension is infectious – toxic and this is a secondary hypotension

**Table 2:** Characteristics of the patients with hypotension

Nr.crt/ Annex	Age	Sex	Group HIV	CD4	Left ventricle dysfunction	Other echocardiographic modification
1/2	31	F	C	119	Pattern I of diastolic dysfunction	Hypokinesia
2/5	31	M	C	50	Pattern I of diastolic dysfunction	Hypokinesia VSd=58mm
3/41	50	M	C	94	Pattern I of diastolic dysfunction	Hypokinesia. Pericardia reflux mice SIV=13mm, PP=11mm
4/53	27	M	A	750		Hypokinesia
5/11	61	M	C	31	Pattern I of diastolic dysfunction	FS=27% SIV=11,6mm
6/20	18	F	A	489	Pattern III of diastolic dysfunction	FS=26%
7/22	27	F	B	315	Pattern III of diastolic dysfunction	FS=27%
8/31	31	F	B	350		FS=25% FE=49%
Total =8	35 years old			274,7/mm³		

Objective heart changes

Clinical heart manifestations in HIV infected patients are discreet. 8 patients (13.3% of cases) have revealed the presence of tachycardia and dyspnea from effort and rest.

**Table 3:** Patients with cardiovascular clinical manifestations

Nr.crt/ Annex	Sex	Years old	HIV Class	CD4 mm3	Echocardiographic modifications
1/2	F	37	C	119	Hypokinesia. Pattern I of diastolic dysfunction
2/41	M	50	C	94	Hypokinesia. Pattern I of diastolic dysfunction. SIV=13mm. Little broken pericardium; PP=11mm
3/20	F	18	A	489	FS=26%. Pattern I of diastolic dysfunction
4/22	F	27	B	315	FS=27%. Pattern III of diastolic dysfunction
5/6	M	60	A	250	VSd=57mm; SIV=12,5mm;VD=40mm
6/31	F	31	B	350	FS=25%; FE=49%. Normal diastolic function
7/34	F	24	A	550	FS=25%; FE=50%. Normal diastolic function
8/53	M	27	A	750	Hypokinesia. Normal diastolic function
Total = 8	M=3 F=5	34.5 years old	A=4 B=2 C=2	364.62 /mm3	

The average age of HIV-infected patients showing clinical cardiac changes is of 34.25 years (3 male and 5 female).

Of the 8 patients, 4 were in class A, 2 in class and 2 in Class C of HIV infection.

The immunological status of these patients was relatively good with a lymphocytes CD4 mean value of 364.62 / mm3. The 2 cases classified as class C HIV had a lymphocytes CD4 mean value of 106,5/mm3, with the significance of a severe immune deficiency.

The highest number of patients showing clinical disorders belongs to a class A of HIV infections (4 patients).

We appreciate that in HIV infection, tachycardia is related with the direct action of HIV virus on the myocardium, or the context of infection induced by an immune depression and created by the virus, which favors infections with opportunistic germs. 

Tachycardia could have a clinical significance of myocardial damage – acute.

It is important to remember a study made in 2000 by Neild [**18**] and collaborators, on 30 patients (among who 10 had dilated cardiomyopathy) by measuring heart rate. Heart rate is very low in patients with dilated cardiomyopathy and is increased in HIV infected patients compared with control groups. The study concludes that HIV infection may be associated with severe autonomic dysfunction even if the clinical diagnose of heart disease is not clear.

Heart rate variability is a marker of cardiovascular autonomic tone, which reduces and decreases with the appearance of cardiac dysfunction. Autonomic dysfunction in HIV infection precedes heart failure, and does not occur as determined by heart failure.

Other authors like L. Moldovan and collaborators [**14-16**] showed in a study that tachycardia, heart deafening noise and congestive heart failure are the most common cardiac manifestations found in HIV infected patients examination. 

An Italian study [**6**] showed that detected clinical changes in cardiac left ventricular dysfunction are present in approximately 15% of the investigated patients.

A study made by Becker K [**1**] showed that the cardiac autonomic dysfunction is severe in patients who are in an end-stage - AIDS compared to HIV-infected patients - in first infection stage.

Another study made by Herdy [**8**] and collaborators, on 50 HIV-infected subjects, 15 of them representing 30%, they described clinically and electrically the presence of sinus tachycardia; hypotension being detected in 7 cases, 14%.

A Portuguese study made by Cardoso JS [**2**] and collaborators indicated that the cardiac symptoms are observed at 7.3% of the HIV infected patients.

The pathological anatomical study performed by us, by evaluating the necroptic protocol of the 85 patients dying due to HIV infection, after autopsy, the clinical diagnosis of heart disease has been established for 5 patients and represents 5.88%.

Clinical symptoms, which suggest the cardiac involvement, have gone unnoticed, overshadowed by noisy suffering symptoms of other organs: the nervous system, respiratory system, digestive system.

Mean age of patients with clinical cardiac changes is of 34.6 years.

The most common clinical diagnosis is the heart failure and is set for 3 patients. Of these, 2 cases describe the pathological anatomical changes. In one case with clinical diagnosis of heart failure, no macroscopic pathological anatomic changes are noticed.

Cardiac pathology and clinical diagnosis is recorded as a cause of death in the first case.

It is a patient of 23 years old, a woman, with clinical diagnosis of ventricular fibrillation, cardiac arrest, dying in year 2000, at necropsy, macroscopic hemorrhagic fluid in the pericardium, in an amount of 300 ml is found.

**Table 4:** Correlation between the anatomic pathology diagnostic - clinical diagnosis

Nr.crt./ annex	Sex	Age	Medium	Clinical diagnosis	Anatomo-Pathological Diagnosis
1/14	M	29	U	Acute Myocarditis. Cardiac insufficiency treated with digital-diuretic	Serous acute pericarditis (100ml). Acute myocarditis. Cardiomegaly
2/19	M	36	U	Cardiac insufficiency treated with digital-diuretic	Acute Myocarditis
3/47	F	19	U	Cardiac insufficiency treated with digital-diuretic	-
4/80	F	23	R	Ventricular Fibrillation	Hemopericardium (300ml)
5/81	F	66	U	Myocardial Cardiopathy	Recent Myocardium Failure. HVS
Total=5classes	M=3 F=2	34,6	U=4 R=1	Myocardial Cardiopathy	Recent Myocardium Failure. HVS		

We can say that in the HIV infection, heart clinical manifestations are suitable: tachycardia sinus, moderate dyspnea.

Clinical diagnosis suggesting heart affection, (cardiac problem,) is established for 8 cardiac cases, representing 13.33% of the patients evaluated in clinical studies. 

In a necroptic retrospection study, the clinical diagnosis of cardiac pain was clinically established for 5.88% of patients.

## Conclusions

The clinical presentation of the heart damage early in the disease is very poor, so: myocarditis, pericarditis or valvular endocarditis is rarely diagnosed and therapeutic implication of these diseases is ignored.

Cardiac complications of HIV infection are more common and cause structural and functional damage by complex mechanisms and the direct infection of cardiac tissue, the severity of immunodeficiency, opportunistic infections have an important role.

Due to the clinical picture opacity, cardiac complications are rarely diagnosed and treated, although they are important for patients’ perspective.
